# Linezolid-resistant *Enterococcus faecium* strains isolated from one hospital in Poland –commensals or hospital-adapted pathogens?

**DOI:** 10.1371/journal.pone.0233504

**Published:** 2020-05-26

**Authors:** Beata Krawczyk, Magdalena Wysocka, Roman Kotłowski, Marek Bronk, Michał Michalik, Alfred Samet

**Affiliations:** 1 Department of Molecular Biotechnology and Microbiology, Faculty of Chemistry, Gdańsk University of Technology, Gdańsk, Poland; 2 Department of Clinical Microbiology, Clinical Hospital No 1, Medical University of Gdańsk, Gdańsk, Poland; 3 Medical Center MML, Warsaw, Poland; Universidade Nova de Lisboa, PORTUGAL

## Abstract

One of the most pressing problems of enterococci infections is occurring resistance to linezolid, which is an antibiotic used in the treatment of infections caused by vancomycin-resistant strains (VRE). The main objective of our research was to investigate the relationship of 19 linezolid-resistant *E*. *faecium* isolates from 18 patients hospitalized at Clinical Hospital in Gdansk (Poland). One of the LZD^R^EF was isolated in 2003 (K_2003_), and another 18 were collected from 2013 to 2017. Genotyping with PCR MP method indicated 14 main unrelated genetic profiles and no association with K_2003_ strain. Two isolates with the same genotype and genetically closely related two sub-types (2 isolates for each sub-type) were hospital-derived colonizations of patients. The other unrelated genotypes were discussed in the context of colonization, nosocomial infections, and commensal origin, taking into account prior exposure to linezolid. We determined the presence of a point mutation G2576T in six loci of 23S rDNA. There was also a significant correlation (p<0.0015) between the presence of MIC>32 value and the presence of G2576T point mutation on the sixth *rrn*. We also detected 5 virulence genes for all isolates: *gel*E, *cyl*A, *asa*1, *hyl*, *esp*. Correlation (p≤0.0001) was observed between the presence of *gel*E gene encoding gelatinase and two other genes: *cyl*A and *asa*1 encoding cytolysin and collagen binding protein responsible for aggregation of bacterial cells, respectively. Significant correlation was also observed between *asa*1 and *cfr* genes encoding 23S rRNA rybonuclease responsible for resistance to PhLOPSA antibiotics (*p* = 0.0004). The multidimensional analysis has also shown the correlation between *cfr* gene and GI-tract (*p* = 0, 0491), which suggests horizontal gene transfer inside the gut microbiota and the risk of colonization with linezolid-resistant strains without previously being treated with the antibiotic. The patient could have been colonized with LZD^R^VREF strains which in the absence of competitive microbiota quickly settle in ecological niches favourable for them and pose a risk for the patient.

## Introduction

Enterococci are present in soil, water, on plants, in dairy products and are a natural component of regular gastrointestinal microbiota of vertebrates. They may also occur on skin, in oral cavities or vagina [1]. For many years enterococci have been believed to be relatively pathogenic organisms. According to the reports of the American National Healthcare Safety Network (NHSN) [2], enterococci are the second genus of bacteria that causes nosocomial infections. The issue of enterococcal nosocomial infections has also been observed by EARSS (European Antimicrobial Resistance Surveillance System) in Europe. An increasing role of these microorganisms as etiological factors of nosocomial infections is also discussed in China [3–5]. Apart from *E*. *faecalis* (80–90%), *E*. *faecium* (5–15%) shows the greatest clinical significance. *E*. *faecium* is cultured from patients treated at haematology, oncology, transplant wards, and intensive care units [6–9]. *E*. *faecalis* is associated with increased virulence, whereas *E*. *faecium* often shows signs of multi-drug resistance (MDR) [10, 11]. It is not uncommon that *E*. *faecium* strains exhibit acquired High Level Aminoglycoside Resistance (HLAR) [12] and resistance to glycopeptides (Vancomycin-Resistant Enterococcus, VRE) [13–15]. Proliferation of vancomycin-resistant clones has also begun to pose an issue in a non-hospital setting [16, 17]. Microorganisms acquire genes for antibiotic resistance by means of conjugation using broad host range plasmids, through transposons carrying numerous genes for antibiotic resistance and as a result of point mutations in the sequence of genes encoding important enzymes involved in cell’s vital processes. A new class of antibiotics, oxazolidinones, linezolid in particular, offers an opportunity for treatment and survival of patients infected with Gram-positive bacteria. It soon turned out that VRE strains may also be resistant to linezolid (LZD^R^VREF strains) [18]. Such linezolid resistance usually results from a point mutation of G2576U or G2447U in the V domain of the 23S rRNA subunit which is a part of ribosomes [19, 20]. This leads to inhibition of the catalytic activity of the peptidyltransferase and, consequently, to inhibition of protein biosynthesis.

Mutations associated with resistance to linezolid have been noted not only in *Enterococcus faecium* but also in other bacteria, such as *E*. *faecalis*, *Staphylococcus aureus*, *Staphylococcus epidermidis*, *Staphylococcus haemolyticus* and *Staphylococcus hominis* which are opportunistic pathogens [21–23]. Some authors report that there is a correlation between the number of rDNA gene copies carrying the G2576T mutation (each genome of *E*. *faecium* encodes 6 copies of rRNA) and phenotypic level of resistance to linezolid, suggesting that the latter is primarily determined by the percentage of ribosomes with G2576U-substituted rRNA [24,25]. Mutation in more than one copy of RNA operon likely results from homologous recombination between mutant and wild-type operons due to selective antibiotic pressure [25]. There are also other types of mutations resulting in resistance to linezolid in addition to G2576U mutation within the 23S rRNA subunit. The *cfr* gene located on a chromosome or plasmids is associated with resistance to linezolid [26, 27]. It encodes an RNA methyltransferase that modifies the 23S rRNA subunit which in turn results in resistance to antibiotics that belong to five classes: phenicols, lincosamides, oxazolidinones, pleuromutilins or streptogramin A (the so-called PhLOPS_A_ antibiotics) [28]. He et al. [29] and Wang et al. [30] have described an *optr*A gene (oxazilidinones phenicol transferable resistance) having an impact on elevated MIC for oxazolidinones (linezolid and tedizolid) and phenicols (chloramphenicol and florfenicol). Protein OptrA is a member of the ATP-binding cassette (ABC)-F protein subfamily. The resistance mechanism to linezolid is based on binding the protein OptrA to the ribosome and the release of the antibiotic conferring inhibition of translation apparatus [31]. It has been proven that multiple *optr*A variants are located on different plasmids and mobile genetic elements. *In vitro* conjugation experiments confirmed the transfer of *optr*A plasmids between enterococci of the same and different species [32].

Both *cfr* and *optr*A are often located on plasmids and may be transmitted horizontally. Mutations in the ribosomal L3 and/or L4 protein are also reported [20]. Resistance to linezolid is relatively rare compared to the number of vancomycin-resistant strains. Two programmes monitoring infections with linezolid-resistant strains are currently being conducted: LEADER which gathers data in the US and ZAAPS (Zyvox® Annual Appraisal of Potency and Spectrum programme), operating worldwide. An emerging problem is the simultaneous resistance to vancomycin and linezolid. Ruru Bi et al., made a systematic review of literature and reported that the percentage of resistance to both antibiotics simultaneously, vancomycin and linezolid, among the 147 strains of *E*. *faecium* was 85.8% [33]. Deshpande et al. also described the problem of VRE and LZD^R^VREF based on the SENTRY Antimicrobial Surveillance Program (2008–2016) [34]. The SENTRY Antimicrobial Surveillance Program conducted on 9,034 *E*. *faecium* clinical isolates showed that only 66 of them exhibited linezolid MIC results of > = 4 mg/l. 74% of these 66 strains were linezolid resistant. At the same time, among these 66 strains as many as 78.1% and 79.5% were vancomycin resistant according to CLSI and EUCAST breakpoint criteria, respectively.

It is recommended that linezolid be used solely in treatment of severe cases where other methods failed. There are only a few compounds at our disposal to treat LZD^R^VREF infections, i.e. daptomycin, tigecycline or a combination of streptogramin A and B. Apart from antibiotic resistance, virulence factors are an additional issue encountered during infections with *E*. *faecium* strains. These factors determine their pathogenicity and virulence degree. Therefore, strains with virulence factors cause infections that are more severe than the ones induced by strains without such factors. Virulence factors may be transmitted by phages, plasmids, transposons or on pathogenicity islands. These include cytolysin (hemolysin), gelatinase, serine protease, aggregation factor, adhesins, capsules, surface proteins, peroxide production, hyaluronidase, lipoteichoic acids, DNAse [35–40]. The different surface adhesins called enterococcal LPXTG surface proteins have been extensively studied in enterococci. They are crucial in to host tissue and their interaction with the host’s innate immune system. Acm (adhesin of collagen from *E*. *faecium*) and Scm (second collagen adhesin of *E*. *faecium*) surface proteins belong to collagen-binding MSCRAMMs (a microbial surface component recognizing adhesive matrix molecules), whereas Esp, is involved in biofilm formation [41].

Genetic typing methods, including multilocus sequence typing (MLST), distinguish high-risk enterococci (HiRECCs clonal complex) with multiple virulence factors and resistance determinants. The high-risk enterococci pose a serious issue in nosocomial infections that lead to outbreaks [40, 42].

The aim of the study was to determine whether linezolid-resistant (LZD^R^) *E*. *faecium* strains isolated from patients in our hospital in 2013–2017 were epidemic strains, whether patients were colonized with hospital strains or whether linezolid-resistant strains were associated with their own microbiota. Genetic typing of LZD^R^ *E*. *faecium* strains was performed in order to compare them with the strain K_2003_, which was the first to emerge in the hospital at the same ward. The issue of colonization with linezolid-resistant strains and associated risks were discussed. Furthermore, we have also determined a mutation type resulting in resistance to linezolid using molecular methods and the correlation between (i) the number of copies of mutations in the 23S rDNA gene and the phenotype of resistance to linezolid (MIC); (ii) LZD^R^EF resistance and prior linezolid treatment or combined antibiotic treatment; (iii) virulence profile of *E*. *faecium* strains and LZD^R^EF resistance.

## Materials and methods

### Characteristic of patients and *Enterococcus faecium* strains

19 linezolid-resistant *Enterococcus faecium* isolates collected from 18 patients (one patient with two isolates) at University Clinical Centre in Gdańsk (Poland) were investigated. Confidential information about patients was not available to the authors of the publication. The Bioethics Committee waived the need for consent from all patients from whom bacterial strains were isolated and used in this study. Samples were taken as part of routine procedures by medical personnel who were not involved in the publication. No additional information about the patients was collected for the purpose of the publication except for the clinical diagnosis and antibiotic treatment. The clinical isolates were collected and archived by technical staff in accordance to the hospital procedures and anonymized before further investigation. Decision no. NKBBN/557/2019 was issued by the Local Bioethics Committee at the Medical University of Gdańsk (Gdańsk, Poland). The first LZD^R^VREF isolate in Poland was described in 2003 in the same Hospital and was included for comparative analysis (designated as a K_2003_) [8]. The next isolates were collected from 2013 to 2017 and stored at -80°C until further analysis.

Most of the patients from whom LZD^R^EF strains were isolated have been treated for: acute myeloid leukemia (9 patients), multiple myeloma (2 patients), non-Hodgkin lymphoma (2 patients), MDS-myelodysplastic syndrome (1patient), acute myelofibrosis (1patient), acute lymphoblastic leukemia (1patient) and they were hospitalized in Department of Adult Haematology or Haematology-Transplantology. Other underlying diseases: refractory anemia (patient from the Department of Children’s Haematology) and coronary artery bypass grafting (patient from Cardiac Surgery Ward), one case each. LZD^R^ isolates were cultured from blood (2 isolates), rectum-swab (9 isolates), stool (5 isolates) urine (2 isolates), bedsore swab (1 isolate). The isolates from blood, urine and bedsore were responsible for symptomatic infections and were found to be invasive. The faeces isolates collected from rectal-swab and stool were recognized as non-invasive.

*E*. *faecium* isolates were identified to the species level using the automated VITEK-2 Compact and API system (bioMérieux, Lyon, France). In addition, PCR-direct detection of the *ddl* gene (D-Ala-D-Ala ligase) unique to *E*. *faecium* species (557 bp) was performed according to Dutka-Malen et al. [43]. Details concerning patients (sex and clinical history) and samples are shown in Table 1. Patients from whom linezolid-resistant strains were isolated have previously been treated with various antibiotics: β-lactams (penicillin, piperacillin / tazobactam, cephalosporins II and IV generation), fluoroquinolones (e.g. ciprofloxacinum), carbapenems (e.g. imipenem, meropenem), aminoglycosides (amikacin), macrolides (clindamycin) and glycopeptides (vancomycin).

**Table 1:** Characteristic of patients and strains of *Enterococcus faecium*.

Patient	Sex	Underlying disease	Ward	Isolate number	Specimen source	LZD MIC [μg/ml]	Treatment previously with LZD	Other antibiotics R or S
P1	F	Multiple myeloma	Hematology	1	blood	64	No	VAN/R; GHL/R AMP/R; TEIC/R; CH/R; T/S; TGS/S
P2	F	Acute myeloid leukemia	Hematology	2	rectum-swab	32	Yes	VAN/R; GHL/R; AMP/R; TEIC/R; CH/S; T/S; TGS/S
P3	M	Acute myeloid leukemia	Hematology	3	urine (UTI)	128	Yes	VAN/S; AMP/R; TEIC/S; CH/R T/R; TGS/S
P4	F	Multiple myeloma	Hematology	4	rectum-swab	96	No	VAN/R; GHL/R; AMP/R; TEIC/R; CH/S; T/S; TGS/S
P5	F	Acute myeloid leukemia	Hematology	5	stool	64	No	VAN/R; GHL/R; AMP/R; TEIC/R; CH/R; T/S; TGS/S
P6	F	Acute myeloid leukemia	Hematology	6	stool	256	No	VAN/R; GHL/R; AMP/R; TEIC/R; CH/R; T/S; TGS/S
P7	M	MDS myelodysplastic syndrome	Hematology -Transplantology	7	rectum-swab	32	Yes	VAN/R; GHL/R; AMP/R; TEIC/R; CH/S; T/S; TGS/S
P8	F	Acute myeloid leukemia	Hematology	8	rectum-swab	32	No	VAN/R; GHL/R; AMP/R; TEIC/R; CH/S; T/S; TGS/S
P9	F	Non-Hodgkin lymphoma	Hematology	9	rectum-swab	32	No	VAN/R; GHL/R; AMP/R; TEIC/R; CH/S; T/S; TGS/S
P10	M	Acute lymphoblastic leukemia	Hematology	10	rectum-swab	32	No	VAN/R; GHL/R; AMP/R; TEIC/R; CH/S; T/S; TGS/S
P11	M	Acute myelofibrosis	Hematology	11	rectum-swab	128	No	VAN/R; GHL/R; AMP/R; TEIC/R; CH/S; T/S; TGS/S
P12	M	non-Hodgkin lymphoma	Hematology -Transplantology	12	stool	128	No	VAN/R; GHL/R; AMP/R; TEIC/R; CH/S; T/S; TGS/S
P13	F	Acute myeloid leukemia	Hematology	13	rectum-swab	32	No	VAN/R; GHL/R; AMP/R; TEIC/R; CH/S; T/S; TGS/S
P14	F	Acute myeloid leukemia	Hematology	14	stool	128	Yes	VAN/R; GHL/R; AMP/R; TEIC/R; CH/R; T/S; TGS/S
P15	F	Acute myeloid leukemia	Hematology	15	urine (UTI)	32	No	VAN/R; GHL/R; AMP/R; TEIC/R; CH/S; T/S; TGS/S
P16	M	Refractory anemia	Pediatric Hematology	16	rectum-swab	32	Yes	VAN/R; GHL/R; AMP/R; TEIC/R; CH/S; T/S; TGS/S
				17	blood–catheter (CRBSI)	32		VAN/R; GHL/R; AMP/R; TEIC/R; CH/S; T/S; TGS/S
P17	M	Coronary artery bypass grafting (CABG)	Cardiosurgery	18	bedsore swab	64	No	VAN/R; GHL/R; AMP/R; TEIC/R; CH/R; T/S; TGS/S
P18	M	Acute myeloid leukemia	Hematology	19 (K2003)	stool	32	Yes	VAN/R GHL/R; P/R; AMP/R; TEIC/R; CH/S; T/R; Q-D/S

R—resistance; S—sensitivity; AMP—ampicillin; CH–chloramphenicol; GHL—high concentration of gentamicin; LZD—linezolid; P–penicillin; Q-D—quinupristin-dalfopristin; T–tetracycline; TEIC—teicoplanin; TGC–tygecycline; VAN–vancomycin.

### Antimicrobial susceptibility of *Enterococcus faecium*

Antimicrobial susceptibility tests were carried out using routine methods at the Clinical Microbiology Laboratory of the University Clinical Center in Gdansk (Poland) in accordance with the Clinical and Laboratory Standard Institute guidelines (CLSI). Interpretation of the results of disk tests (AB Bio disk) was carried out in accordance with the manufacturers' procedures and the current EUCAST v 10.0 (2020) recommendations (The European Commitee on Antimicrobial Susceptibility Testing (http://www.eucast.org/). Minimum inhibitory concentrations (MICs) for vancomycin and linezolid were determined using E-test strips (bioMèrieux SA, France) on MHA plates incubated at 35±1°C for 18±2 hours. E-test procedures were performed according to the manufacturer’s guidelines. The strain with MIC>4 μg/ml was classified as resistant to linezolid. In addition, resistance to chloramphenicol and tetracycline were determined by disc diffusion method with reading by automatic BIOMIC Microbiology System. EUCAST v 10.0 recommendations for *E*. *faecium*, do not include assays for chloramphenicol nor tetracycline, therefore the results are qualitative and based solely on breakpoints without specific MIC, according to CLSI M100 recommendations 30th Edition.

### DNA extraction

Cultures were grown overnight in 3 ml of brain heart infusion (BIOCORP, POLAND) broth with paper linezolid (10 μg) disc (BD BBL^™^ Sensi-Disc antimicrobial susceptibility) at 37°C for 24 h. Chromosomal DNA for genotyping was extracted from 1.5 ml culture by using genomic DNA kit (EXTRACTME Genomic DNA KIT, BLIRT S.A., Gdansk, Poland) according to the manufacturer's instruction for enterococci. 100 μl of cell suspension was supplemented with 20 μl of lysozyme solution (1mg/ml) and incubated for 30 min at 37°C. Efficiency of DNA isolation determined by NanoDrop Spectrophotometer ND-100 (Thermo Fisher Scientific, Wilmington, USA) with a concentration range from 20 ng/μl to 40 ng/μl. Total DNA obtaining by an alkaline lysis extraction method and isopropanol precipitation (DNA concentration: 10–20 ng/μl) was used to detect *optr*A and *cfr* genes.

### Genotyping of *E*. *faecium* strains

Genotyping was carried out for 19 isolates, including 18 LZD^R^EF isolates collected in the period from 2013 to 2017 and one LRVREF *E*. *faecium* strain isolated from the same hospital but in 2003 (control strain K_2003_). Molecular characterization of clonal identity of all isolates was performed by means of PCR melting profiles (PCR MP) method as has been described previously [9] with the following modification. We used the *Eco*RI restriction enzyme at the step of digestion of total genomic DNA instead of the *Hind* III. All sequences of oligonucleotides for adapters and primers are included in S1 Table. The reaction profile with the denaturation temperature in the PCR cycles was the same as described in the work of Krawczyk et al. [9]. Polyacrylamide gel electrophoresis (6%) in 1xTBE buffer for the separation of DNA fragments was prepared and then was stained with ethidium bromide. Subsequently, FPQuest TM software (BioRad; version 4.5) for comparative analysis of the DNA patterns was used. Similarities between genetic fingerprints were calculated using Dice band-based similarity coefficient (SD) with UPGMA method.

### Detection of resistance genes by PCR

#### Confirmation of the VanA phenotype by PCR

The presence of *van*A and *aac*(6')-*aph*(2'') genes that are responsible for VanA fenotype with resistance to high concentration of gentamicin (gentamycin HL) were detected by simplex PCR from total DNA using primers to PCR and thermocycling conditions designed by Yean et al. [44] (S1 Table).

#### Detection of *cfr*A and *optr*A genes by PCR

All LZD^R^EF isolates were also tested for the presence of *cfr*A and *optr*A genes by PCR, using total DNA, according to procedure as described previously [30, 45, 46]. The primers sequence are given in the S1 Table. Due to the expected long PCR product (~1400 bp) for *optr*A gene Hypernova DNA polymerase (DNA Gdańsk, Blirt S.A, Poland) was applied. The clinical isolates of *E*. *faecium* and *E*. *faecalis* from the laboratory collection were used as positive controls for the *cfr* and *optr*A genes, respectively.

### Molecular detection of the G2576T mutations and evaluation of heterozygosity in *rrn* operons

#### PCR-RFLP

PCR-RFLP method for detection of G2576T mutation was applied according to Ruggero et al. [47]. The 662-bp amplicons were digested using *Nhe*I restriction endonuclease (New England Biolabs) at 37°C for 2.5 h. The DNA fragments were separated in 1.5% agarose gel (HiREs Grade, BioLine) and stained with ethidium bromide.

#### Real-time PCR

For detection of G2576T mutation among *E*. *faecium* strains exhibiting resistance to linezolid we used HRM-real-time PCR method using MyGo Pro Real-Time PCR System (MG-P, IT-IS International LTD).

PCR amplification (primers in S1 Table) and high resolution melting (HRM) analysis of 197 bp amplicons were performed using Sensi FAST^TM^ HRM Kit (BioLine GmbH, Germany) in 20μl final reaction mix. Reaction mix composition was according to manufacturer's kit, only DNA and magnesium ions concentrations were optimized. 3–6 ng of DNA and 3.5 mM of Mg^2+^ were used per reaction.

The PCR profile was: one cycle 300 s at 94°C; 30 cycles of 94°C for 10 s, 53°C for 10 s, 72°C for 10 s; one cycle 10 s at 95°C. HRM at first step—64°C for 15 s [ramp 4°C/s], HRM at final step 95°C for 1s [ramp 0.05°C/s], one cycle 45°C for 15s [ramp 5°C/s]. *MyGo Pro* Software 2.0 was used to analyze the melting temperature in order to detect the G2576T mutation for each individual isolate and identification of the groups with or without mutation.

### Detection of the number of 23S rRNA genes with the G2576T mutation

Six copies of 23S rDNA gene for each *E*. *faecium* strain were separately amplified by PCR with Hypernova DNA polymerase (DNA Gdańsk, Blirt S.A, Poland). One universal forward primer sFlr100 [47] and six reverse primers [48] Efrev: C1, C2, C3, C4, C5, C6 specific to six copies of 23S rDNA genes were used (S1 Table). Amplification conditions were as follows: denaturation for 5 min at 95°C (one cycle), 30 cycles of denaturation for 30 s at 95°C, annealing for 1 min at 49.4°C for C1 primers, at 48.5°C for C3 primers, at 56.3°C remaining C2, C4, C5 and C6 primers; extension for 150 s at 72°C, and a final extension of 6 min at 72°C.

Then each copy of the amplified gene was digested by *Nhe*I restriction enzyme. In the case of G2576T mutation, characteristic fragments for each copy were generated (Table 2) and visualized in 1.2% agarose gel separation method.

**Table 2:** Amplification of individual copies (C1-C6) of the 23S rDNA and detection of G→T mutation by *Nhe*I digestion.

copy 23S rDNA gene	C1	C2	C3	C4	C5	C6
product PCR [bp]	1223	1833	1346	1337	2590	2175
*Nhe*I restriction fragments	656, 567	1266, 567	779, 567	770, 567	2023, 567	1608, 567

### PCR detection of virulence factors

Virulence genes coding for enterococcal surface protein (*esp*), aggregation substance (*asa*1), cytolysin (*cyl*A), hyaluronidase (*hyl*) and gelatinase (*gel*E) were detected using specific primer sequences presented in S1 Table. Hypernova DNA polymerase (DNA Gdańsk, Blirt S.A, Poland) was used for amplification. The PCR reactions were carried out in an Eppendorf thermocycler according to the following time-temperature profile: denaturation for 5 min at 95°C (one cycle), 30 cycles of denaturation for 45 s at 94°C, annealing for 45 s at 63°C for *esp* gene, at 56°C for *asa*1, *cyl*A and *hyl* genes, at 53°C for *gel*E gene; elongation step for 1 min at 72°C, and a final extension for 5 min at 72°C.

### Statistical analysis

Graphical statistical analysis of the correlation parameters was carried out with the Past v. 3.22. software [https://folk.uio.no/ohammer/past/] [49]. P-values using the Fisher’s exact test were calculated using free Statistics Software, Office for Research Development and Education, version 1.2.1, URL was used, (http://vassarstats.net/tab2x2.html).

## Results and discussion

Nowadays acquired resistance mechanisms are of greatest clinical and epidemiological importance. These include high level aminoglycoside resistance (HLAR), glycopeptide resistance (VRE) and resistance to linezolid (linezolid-resistant enterococci–LZD^R^EF) [7, 16, 50]. Enterococci are part of the commensal flora of the human gastrointestinal and genitourinary tracts. They are mostly susceptible to antibiotics. However, Haghi et al. presented a report about high frequency of vancomycin resistance enterococci isolated from faecal samples of healthy people [51]. They are considered to be carriers of vancomycin resistance strains. We suspect that resistance to linezolid may also appear among commensal strains in gut, even without the patient being treated with linezolid. The human GI tract is a convenient environment for bacterial evolution and acquisition of antibiotic resistance. Antibiotic resistance among enterococci in GI tract is the result of genetic mutations, horizontal gene transfer, or the expression of resistance genes from other strains. Biofilms are found in the large intestine and are considered the ideal environment for horizontal gene transfer due to the high bacterial density and the physical protection for cells [52]. In another study Salyers et al. hypothesized that human intestinal bacteria can serve as a reservoirs for antibiotic resistance genes [53]. Montecalvo et al. indicated that enterococcal infections are most frequently caused by the patient’s own commensal flora [54]. The resistance can be transferred to commensal intestinal microbiota or transient bacteria which are not colonizing the gut.

Even with limited or no antibiotic use, antibiotic resistance determinants can be stably maintained within the bacterial population. Commensal strains that have been transformed into pathogens in the human gut are often misinterpreted as hospital-derived strains (hospital colonization or nosocomial infection). These commensal strains can spread in the hospital environment and quickly become hospital strains. Furthermore, the patient may be colonized immediately before or long before the infection. The current lack of LZD^R^VREF detection in the hospital environment, does not mean that there is no risk of infection. Colonization and infection with linezolid-resistant strains to linezolid is possible after antibiotic therapy, during which other bacteria are eliminated. As such, the then emptied ecological niches can quickly be colonized by enterococci in the absence of competition.

Haematooncological patients undergoing long-term hospitalization, cancer treatment or broad-spectrum antibiotic therapy constitute a significant group of patients in our study from whom LZD^R^EF strains were isolated. In such population, the damaged gastrointestinal and oral mucous membranes are damaged as well as immunosuppression associated with neutropenia and reduced IgA foster development and spread of infections. *E*. *faecium* infections in this group of patients may originate from their own regular gastrointestinal microbiota. Apart from wild-type strains, enterococci from colonized gastrointestinal, oral and vaginal mucous membranes are responsible for endogenous infections [8, 15]. These strains become multi-drug resistant (MDR) as a result of broad-spectrum antibiotic therapy. Colonization with MDR strains in this group of patients is associated with high risk of UTI and may also lead to translocation of bacteria from the GI tract into blood vessels, as described in case of *Escherichia coli* [55–57]. Characteristics of our patients and strains are presented in Tables 1 and 3.

**Table 3:** Percentage share of individual LZD^R^ isolates by the type of clinical sample, distribution of virulence-associated genes and drug-resistance in *Enterococcus faecium* depending on clinical samples.

No. (%) of clinical samples	Virulence-associated genes					Antimicrobial agents						
	*esp*	*asa*1	*cyl*A	*gel*E	*hyl*	AMP	VAN	TEIC	LZD	GHL	CH	T
rectum swab 9 (47.4)	9	7	7	6	7	9	9	9	9	9	0	0
stool 5 (26.3)	5	4	2	3	4	5	5	5	5	5	3	1
blood 2 (10.5)	2	1	1	1	1	2	2	2	2	2	1	0
urine 2 (10.5)	2	2	2	2	2	2	1	1	2	1	1	0
bedsore swab 1 (5.3)	1	0	1	1	1	1	1	1	1	1	1	0
Total clinical samples 19 (%)	19 (100)	14 (73.7)	13 (64.4)	12 (63.1)	15 (78.9)	19 (100)	18 (94.7)	18 (94.7)	19 (100)	18 (94.7)	6 (31.6)	1 (5.3)

*esp—*enterococcal surface protein; *asa*1*—*aggregation substance; *cyl*A*—*cytolysin; *gel*E—gelatinase; *hyl—*hyalorunidase; AMP—ampicillin; VAN—vancomycin; TEIC—teicoplanin; LZD—linezolid; GHL—high concentration of gentamicin; CH—chloramphenicol; T—tetracycline.

Most isolates originated from rectal swabs and stool (n = 14, 73.7%). These strains from gastrointestinal tract were treated as non-invasive ones (without symptoms of infection). The isolates from blood, urine and bedsore were responsible for symptomatic infections and they were found to be invasive (n = 5, 26,3%).

In two haematological patients isolates originated from blood cultures. One patient (P1) had sepsis diagnosed and the other one (P16) had LZD^R^VREF *E*. *faecium* found at first in anus and then in blood drawn from intravascular (IV) catheters. As reported by various research centres, nosocomial bacteraemias account for 55–75% cases of the total number of bacteraemia episodes [58, 59]. IV catheters often become a gateway for infections (CRBSI—catheter-related bloodstream infection), in particular at haematology and oncology wards, and at intensive care units. Urinary tract infection (P3, P15) was noted in two patients with acute myeloid leukaemia (AML). Such infections are fostered by immunosuppression, post-transplant catheterization time, broad spectrum antibiotic therapy and presence of intravascular catheters. The hospital setting is particularly responsible for selective pressure and facilitates formation of multi-drug resistant enterococci strains, including LZD^R^VREF strains, due to excessive use of i.a. cephalosporins, aminoglycosides and fluoroquinolones, and above all, glycopeptides and oxazolidinones. Other risk factors for nosocomial LZD^R^VREF infections include: duration of hospitalization, contact with patients colonized and infected with LZD^R^VREF, the same hospital staff (including a nurse) who takes care of patients, severity of underlying disease, recent surgery and immunosuppression.

### Epidemiological studies based on genotyping of *E*. *faecium* strains

**Fig 1:**
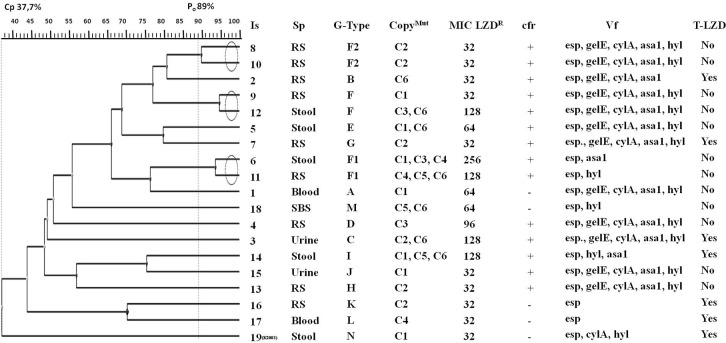
The phylogenetic relationships for clinical LZD^R^EF isolates, characteristics of linezolid resistance and virulence profiles. The scale value represents 0.06 parameter substitution per site. Legend: Is—isolate; Sp—specimen;RS—rectum swab; SBS—bedsore swab; G-Type–genotypes designation as (A-N); CopyMut—copy number with a mutation; *cfr—*gene coding for 23S rRNA methyltransferase; Vf—virulence factors: *esp*—enterococcal surface protein; *asa*1—aggregation substance; *cyl*A—cytolysin; *gel*E—gelatinase; *hyl—*hyalorunidase; T-LZD—treatment previously with linezolid.

*E*. *faecium* isolates associated with hospital outbreaks and recorded in Poland using the MLST (Multi-Locus Sequence Typing) are representatives of 17/18 and 78 lineages that are widespread throughout the world and in Europe [60]. MLST is a technique based on sequencing of housekeeping genes and is used as a tool in global and long-term epidemiological analysis [61]. *E*. *faecium* falling into clonal complex 17 (CC-17) are very often resistant to vancomycin. A method with high discriminatory power seems appropriate for studying bacterial strains from one hospital; thus PCR MP (Polymerase Chain Reaction Melting Profile) was used for epidemic typing of LZD^R^EF strains instead of MLST. PCR MP has been for many years used by our research team, also to type enterococci [9]. It has higher discriminatory power and makes it possible to distinguish epidemic from endemic strains, and genotypes that are not associated with an outbreak. Eighteen LZD^R^EF from October 2013 to June 2017 and a strain K_2003_ resistant to vancomycin and linezolid (LZD^R^VREF), which was the first one to emerge in this hospital in 2003 [8] were genotyped. The patterns with the Dice coefficient above 0.89 were assigned to the same genotype. Fourteen the main genotypes were identified and marked with letters from A to N (Fig 1).

The isolates Is9 and Is12 were considered to be genetically identical (*Pi* 94.5%), and marked with the F genotype, while the isolates 6, 11, 8 and 10 were considered to be genetically closely related to F. They were designated as the sub-types F1 and F2, respectively. They have been isolated from November 2013 to February 2014. Although faecal samples were recognized as a non-invasive, we believe that the patients with hospital colonization can be a potential risk factor for the nosocomial infection and hospital outbreak. In order to limit the spread of LZD^R^EF strains, even these patients with colonization should be monitored. Other isolates, even those isolated from patients with UTI or bloodstream infections were classified as non-epidemic ones. None of the18 isolates was related to the LZD^R^VREF strain from 2003. The existence of such a variety of genotypes among 19 studied isolates may be the result of horizontal gene transfer (HGT). The enterococci are resistant to environmental factors, enabling them to survive in hospital conditions up to several months, and their genomes may gradually change. Another explanation for the diversity of LZD^R^EF genotypes is their commensal origin from the gastrointestinal and genitourinary tracts of patients [62, 63].

### Characteristics of antibiotic resistance

Glycopeptides, such as vancomycin and teicoplanin, are compounds that have effect on the bacterial cell wall. VanA and VanB phenotypes are of the greatest clinical significance. The genes responsible for encoding VanA and VanB phenotypes are located on transposable elements, i.e. transposons, and are easily spread between bacterial cells. VanA phenotype occurs in enterococci as a result of their exposure to both of vancomycin and teicoplanin. Vancomycin resistance (MIC>256 μg/mL) and teicoplanin resistance (MIC>32 μg/mL) were exhibited by 18 out of 19 isolates studied. VanA phenotypes were confirmed by detection of *van*A gene in PCR that resulted in a 931 bp product. Isolate 3 was an exception as it exhibited phenotypic resistance to linezolid with concomitant susceptibility to vancomycin. No amplification product of the *van*A gene was obtained for this isolate. Eighteen LZD^R^EF isolates also had HLGR (high-level gentamicin resistance) phenotype (MIC>128 μg/mL according to EUCAST; EUCAST documents www.escmid.org/research_projects/eucast/). The high-level gentamicin resistance is an effect of a bifunctional enzyme with acetyl phosphatransferase (Aac(6’)-Aph(2”) activity that conditions cross-resistance to all aminoglycosides (except for streptomycin). Presence of *aac(6')-aph(2'‘)* gene was confirmed in PCR. The 156 bp amplification product was obtained as anticipated.

Furthermore, susceptibility to chloramphenicol and tetracycline was determined. Merely 15% of *E*. *faecium* strains isolated from European patients turned out to be resistant to chloramphenicol [64]. In our studies 31.6% and 5.3% of isolates were resistant to chloramphenicol and tetracycline, respectively. Isolate 3 was the only one to exhibit resistance to both chloramphenicol and tetracycline. Antibiotic susceptibility profiles for LZD^R^EF strains are shown in Tables 1 and 3, with specimen sources listed.

### Phenotypic resistance to linezolid

Prior studies suggest that linezolid resistance may be associated with long-term treatment with various antibiotics, vancomycin in particular [59]. Linezolid is used to treat infections caused by *E*. *faecium* isolates resistant to glycopeptides or if a patient is glycopeptide-intolerant. Case-controlled studies with patients treated with linezolid conducted by Pai et al. showed that resistance to linezolid developed not only when patients received various antibiotics, but primarily when they were treated with linezolid over a long period of time (approx. 30 days) [50]. The authors have also observed that LZD^R^EF strains developed even if patients had no history of exposure to linezolid.

When a strain resistant to linezolid and vancomycin developed in a patient P1 with sepsis in October 2013, isolation procedures for hospitalised patients were initiated in subsequent days and months at the same hospital. Eighteen isolates from 17 patients hospitalised from October 2013 to December 2017 showed high resistance to linezolid (MIC = 32–256 μg/mL) and concomitant resistance to vancomycin and teicoplanin (except for one linezolid-resistant and vancomycin-susceptible isolate 3). All patients were previously treated with various types of antibiotics: β-lactams, fluoroquinolones, carbapenems, aminoglycosides, macrolides and glycopeptides. Unsurprisingly, enterococci isolated from these patients were resistant to all groups of antibiotics listed, including linezolid. Only *E*. *faecium* strain isolated from patient 3 showed no resistance to vancomycin, with relatively high resistance to linezolid (MIC = 128 μg/mL), suggesting that susceptibility to vancomycin does not exclude resistance to linezolid.

In our studies 6 patients had a history of treatment with linezolid, which may suggest that resistance to this antibiotic results from selective pressure. But 12 other patients were not treated with linezolid; yet strains isolated from them were highly resistant to linezolid. This group consisted primarily of patients with blood cancers (except for one patient hospitalised at the cardiosurgery ward) who underwent chemotherapy and aggressive antibiotic therapy, along with immunosuppressive treatment in some cases (2 patients from the transplantation ward). During such treatments sensitive microbiota species are eradicated and only the most adapted ones survive. In such scenario endogenous gastrointestinal microbiota that acquired resistance to linezolid may overgrow. LZD^R^EF strains occurring in patients’ gastrointestinal tract may pose a risk for themselves, leading i.a. to urinary tract infections (e.g. P3, P15) or CRBSI (P16). At the same time, such patients are a reservoir of LZD^R^EF strains which may spread and pose a risk of nosocomial infections in a hospital setting and if appropriate isolation procedures are not followed. This fact may be demonstrated by relatively close genotypic relationship between specific strains, even though they were isolated from different patients at a one- or two-month interval (Fig 1).

LZD^R^EF were also isolated from urine of two patients with UTI and from blood of one patient with sepsis. They were not classified as endemic nosocomial strains based on genotyping, which may suggest that urinary tract infection or sepsis may result from prior colonization or transfer from the gastrointestinal system. In case of sepsis, blood infection due to endogenous transfer cannot be excluded.

LZD^R^EF strain susceptible to vancomycin was isolated from urine of patient 3. Genetic typing did not indicate genetic and epidemic relationship with other LZD^R^EF isolates. This patient was previously treated with linezolid. He was likely a carrier of the linezolid-resistant strain that developed due to prior treatment. There is no relationship between prior treatment with vancomycin and resistance to linezolid as described in many other cases.

### Origin and transmission of LZD^R^EF isolates

We presented hypotheses explaining the origin of linezolid-resistant strains in one hospital in Gdańsk. LZD^R^EF isolates were divided in the two main groups. The first group included 14 non-invasive isolates from faecal samples. 6 out of 14 isolates (Isolates numbers: 6, 8, 9, 10, 11, 12) were found to be closely related suggesting a hospital colonization with a common origin. These isolates were from patients without symptoms of infection and without prior exposure to linezolid. The remaining 8 isolates were found to be non-clonal origin. 5 of 8 isolates were from patients with prior exposure to linezolid (Is: 2, 7, 14, 16, 19). They were considered to be commensals-derived strains (endogenous enterococci from GI tract) as a consequence of exposure to antibiotic or colonization by hospital-adapted LZD^R^ enterococci. 3 other isolates recovered from patients without prior linezolid therapy (Is: 4, 5, 13) can be hospital-adapted enterococci or have commensal origin.

The second main group contains 5 unrelated invasive isolates (Is: 1, 3, 15, 17, 18) derived from blood, urine and SBS. Two isolates (Is: 3, 17) from urine and blood (CRBS) were recovered from patients with prior exposure to linezolid. We do not exclude endogenous infections. Three other isolates in this group were derived from blood-stream infection, urine and swab bedsore (Is: 1, 15, 18) from patients without exposure to linezolid. They were considered as hospital infections. The hypothesis of the origin of LZD^R^EF and their transmission between patients and the hospital environment is shown in S1 and S2 Figs.

### Detection of mutation associated with resistance to linezolid

Oxazolidinone antibiotics show mainly bacteriostatic activity, inhibiting protein synthesis by blocking the initiation of translation. The G2576U mutation in 23S rRNA is the most common cause of resistance to linezolid. Phenotypic tests weakly correlate with presence or absence of G2576U mutation, therefore it was decided to use molecular tests [65].

The G2576T mutation in the 23S rDNA gene sequence generates a new restriction site for *Nhe*I enzyme. As such, PCR/RFLP is most often used to detect polymorphism leading to resistance to linezolid. In our study 662 bp amplification products were digested with an excess of *Nhe*I enzyme (20U), yielding 570 and 100 bp fragments and thus confirming presence of the G2576T mutation. Apart from two fragments, the figure still shows a 662 bp fragment, which may indicate heterozygosity in terms of mutations in the 23S rDNA copies. Identical results were obtained for all isolates that exhibited phenotypic resistance to linezolid. Amplification product which did not undergo enzymatic digestion along with the amplification product of the 23S rDNA gene fragment from the linezolid-susceptible strain (without mutation) were used as controls (S3 Fig).

**Fig 2:**
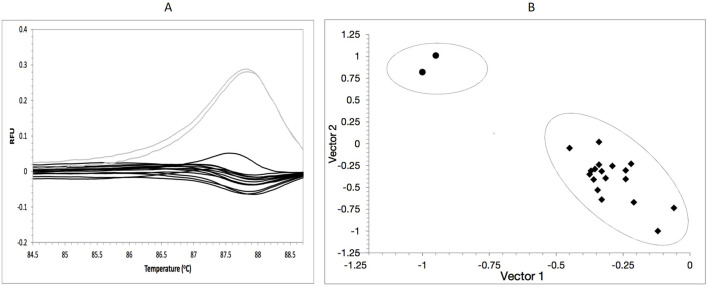
Real-time PCR and high-resolution melt analysis for detection of point mutations responsible for linezolid-susceptible. (A) High-resolution melting profiles for 23S rDNA fragment gene/s with or not G2576T mutation/s. Legend: grey colour–wild strains of *E*. *faecium*; black colour–strains with G2576T mutation/s. (B) A graph generated base on HRM—real time PCR, showing the assignment of the strains to clusters, each cluster is surrounded by an ellipse corresponding to a different genotype, with (bigger cluster) or not (smaller cluster) G2576T mutation.

Heterogeneous population of *E*. *faecium* strains is highly probable. In addition to resistant ones, there are also susceptible ones, in particular if selective pressure factor is removed, e.g. when treatment with linezolid is discontinued. It is reported that full reversion to wild-phenotype or spontaneous reversion for particular mutated copies is possible in the absence of linezolid, which is sometimes difficult to detect with the sequencing method [66]. Another course of treatment with linezolid leads to the reappearance of highly resistant isolates, even if it is a short-term therapy. Real-time PCR was applied in order to ensure detectability of mixed populations, with G2576T polymorphism or wild-type alleles. As resistance to linezolid is associated with substitution of a single nucleotide, it is necessary to apply the high resolution melting curve analysis, which allows for detection of a difference in melting temperature of amplicons below 1°C. A 196 bp fragment was amplified (S1 Table). Eighteen mutated strains from a period of 2013–2017 and 2 non-mutated control strains were tested. Analysis of melting temperature (Tm) of amplification products obtained confirmed presence of polymorphic sequences relative to the two control strains (Fig 2A). The amplification products without mutation showed melting temperature of 88°C, while amplicons with point mutation showed melting temperature lower by approx. 0.3–0.5°C. Furthermore, analysis of differential and normalized curves made it possible to differentiate linezolid-resistant strains from susceptible (control) ones based on the difference in RFU values as a temperature function. The HRM analysis of all linezolid-resistant strains (1–18) and two linezolid-susceptible strains revealed two separate genotypic clusters (Fig 2B).

The smaller cluster consists of control strains without mutations in the 23S rDNA genes and the second one consists of strains with G2576T mutation detected. Using real-time PCR, it is easy to distinguish strains with and without mutation and demonstrate that *E*. *faecium* population is heterogeneous in terms of resistance to linezolid. Schnitzler et al. (2011) reported that strains susceptible to linezolid showed MIC>256 μg/mL and G2576T mutations in 6 copies of 23S rDNA after 50 *in vitro* passages [67]. Strains studied were subjected to *in vitro* selective pressure only for 24 hours in linezolid culture, hence real-time PCR amplification ruled out population heterogeneous in terms of linezolid resistance. Enterococcal strains may form a mixed population *in vivo*; thus not only mutated enterococci but also revertants can be directly detected in the specimen.

### Detection of the G2576T mutation in individual copies of the 23S rDNA gene of the *rrn* operons

The real-time PCR technique does not yield information about the number of copies with single nucleotide polymorphism (SNP). Therefore, a test to detect the G2576T mutation in individual copies of the 23S rDNA gene of the *rrn* operons was developed. The DNA fragment for each copy of the 23S rDNA gene was amplified separately. One universal FORWARD sFlr100 primer (the same for each copy) and a specific primer (REVERS) to detect each copy of the 23S rDNA (C1-C6) were used. The amplification products for C1-C6 were afterwards separately digested with the *Nhe*I restriction enzyme. The G2576T mutation in the 23S rDNA gene is present if PCR products are digested (Table 2).

Seven isolates (3, 5, 6, 11, 12, 14, 18) carried more than one mutated copy of the 23S rDNA gene. Furthermore, the mutation was most often present in C6 (7 isolates), C1 (7 isolates) and C2 (6 isolates). Fig 1 lists mutated copies for individual *E*. *faecium* isolates. Presence of 3 copies of the 23S DNA with a mutation is typical of genotype F1 determined by PCR MP, common to isolates 6 and 11. However, they occur in different copies. C1, C3 and C4 are mutated in isolate 6, and C4, C5 and C6 are mutated in isolate 11.

Isolates 9 and 12 are also in close genetic relationship with each other, but they differ in the number of G2576T mutations in different copies (isolate 9 has one mutated copy (C1), isolate 12 has two mutated copies (C3 and C6)). The same genotype F, as determined by genetic typing, is not equivalent to the same mutated copy of the 23S rDNA gene. According to our hypothesis the G2576T mutations could appear in different copies and in the different number as an independent process of evolution for each strain after patient colonization.

Isolates 6, 11, 12 with G2576T mutations in 2–3 copies of the 23S rDNA gene have been cultured in samples obtained from patients who were not previously treated with linezolid. Therefore, the impact of selective pressure of linezolid in this case is out of question. As such, mutations could be related to prior treatment with numerous antibiotics, including vancomycin.

Rugerro et al. (2003) and Marshall et al. (2002) suggest the mutated gene dosage effect on LZD^R^EF MIC [47, 68]. Isolates in our study were highly resistant to linezolid. Most isolates had MIC of 32 μg/mL and carried single-copy mutations related to C1 and to C2. In contrast, isolates with more than one mutated copy and MIC = or >64 μg/mL often exhibited mutations in C6. Isolates with more than two G2576T mutations showed relationship with C3/C4/C5 and often exhibited resistance to chloramphenicol. It is not possible to clearly confirm that MIC increases with the number of copies with G2576T mutations, although in 5 isolates high MIC correlates with increased number of mutated copies (isolates 3, 6, 11, 12, 14). Fig 1 presents mutations in individual copies of the 23S rDNA genes relative to MIC.

### Detection of *cfr* and *optr* genes as a way to find other mechanisms of linezolid resistance

Additionally, the aim of this study was to determine the relationship not only between elevated MIC and the number of mutated copies of the 23S rDNA gene but also between the presence of the *cfr* or *optr* gene. The *cfr* gene encodes an RNA methyltransferase responsible for modification of the V domain of the 23S rRNA subunit, which may lead to resistance to oxazolidinones [26, 27, 28]. The *cfr* gene is often located on a plasmid and may be horizontally transmitted between *E*. *faecium* strains and staphylococci or non-faecal streptococci [28]. This mechanism of resistance to linezolid cannot be excluded in case of patient P10 who was colonized by a LZD^R^VREF strain which underwent G2576T point mutation and was a carrier of the *cfr* gene.

The *cfr* gene was detected in 14 isolates, but there was no correlation between its presence and MIC. Isolates without the *cfr* gene showed MIC LZD^R^ of the same value as isolates with this gene.

On the other hand, the *optr* gene had impact on elevated MIC for oxazolidinones and resistance to phenicols (chloramphenicol and florfenicol) [29, 30]. Six isolates demonstrated resistance to chloramphenicol, therefore the search for the *optr* gene was suggested. Neither isolates resistant to chloramphenicol nor the ones susceptible to it carried the gene. Furthermore, isolate 3, which was susceptible to vancomycin but resistant to linezolid, was the only one to exhibit resistance to both chloramphenicol and tetracycline. Resistance to chloramphenicols results from the presence of mobile genetic elements, such as plasmids, transposons or integrons with gene cassettes, but it may also be caused by reduced permeability of outer membranes [69].

### Detection of virulence factors

Enterococci acquire new properties due to plasticity of their genome, enormous adaptive capacity and constant pressure to adapt to new conditions in hospital setting. They may survive in such conditions for many years [14]. Enterococci, which have virulence factors, are potentially capable of inducing an infection with a more severe course than strains deprived of them. Therefore, another aim of the study was to determine the virulence degree of strains resistant to linezolid and to assess risk for patients in case of infection/colonization. In order to determine virulence degree of LZD^R^EF strains, the following fragments of 5 genes relevant for virulence were amplified: *esp*–encoding Esp surface protein that affects ability of *E*. *faecium* to create biofilm, *cyl*A–with the function of cytolysin/haemolysin, *gel*E–encoding gelatinase and responsible for decomposition of collagen, *hyl*–encoding hyaluronidase and participating in bacterial spread throughout the organism, *asa*1 –encoding collagen-binding protein which allows enterococci to colonize the host’s organism [39, 41, 66].

The majority of LZD^R^EF strains studied had *asa*1, *cyl*A, *gel*E, *hyl*, *esp* genes that play a significant role in adherence to intestinal epithelium, as well as epithelium of urinary tract or in transmission to blood vessels (Fig 1). Table 2 shows percentage share of individual virulence factors and resistance to antibiotics, including specimens from which LZD^R^EF bacteria have been isolated. All LZD^R^EF strains carried the *esp* gene. According to literature, this gene is found mainly in strains resistant to numerous antibiotics and strains isolated from patients with nosocomial infections, whereas serine protease (Esp) plays the most significant role as a factor allowing enterococci to colonize and infect the urinary tract [70]. The set of strains used in our study included the ones responsible for UTI, sepsis or colonization of the GI tract; the *esp* gene was found in all of them. The *hyl* gene was the second most common virulence factor found in approx. 79% of strains. Hyaluronidase coded on chromosomes in *E*. *faecium* indicates increased virulence of this bacterial species and contributes to destruction of connective tissue. A hyaluronidase-encoding gene was found in numerous vancomycin-resistant *E*. *faecium* strains [71]. Our studies also revealed that it may be of significance for linezolid-resistant enterococci.

The *asa*1 gene (approx. 74%) was the third most frequent gene, followed by *cyl*A (approx. 64%) and *gel*E (approx. 63%). The *asa*1 and *cyl*A genes are located on the conjugation plasmids and may be easily transmitted between bacteria [72]. The *asa*1 gene product, an aggregating substance, increases bacterial adherence to renal tubule cells, endocardial cells and intensifies internalization by intestinal epithelial cells [34]. This virulence factor makes it easier for LZD^R^ strains to colonize both the GI tract and urinary tract or poses a risk for patients who underwent cardiac surgery, giving the bacteria a better chance of successful colonization of their ecological niche. In contrast, CylA toxin exhibits cytolytic activity, causing changes in the integrity of the cell membrane (e.g. haemolysis of erythrocytes) [73]. Cellular lysis is not the only effect of this toxin. Cytolysins also interfere with functioning of the intracellular metabolism, as they attack immune cells (leukocytes, macrophages) and consequently disturb their function. Strains with these virulence factors may be particularly dangerous for haematological patients. The *gel*E gene encoding metaloendopeptidase is located on a chromosome and plays an important role in the pathomechanism of enterococcal infections, particularly during invasion, causing hydrolysis of elastin or collagen.

Although mostly *E*. *faecalis* strains show increased virulence, while *E*. *faecium* exhibit mainly multi-drug resistance [11], in this case the majority of LZD^R^VREF is both resistant to numerous commonly used antibiotics and rich in virulence factors, making them dangerous for patients and a risk of epidemiological transfer.

### Correlation between antimicrobial resistance, virulence and clinical aspect of *Enterococcus faecium*–multidimensional analysis

**Fig 3:**
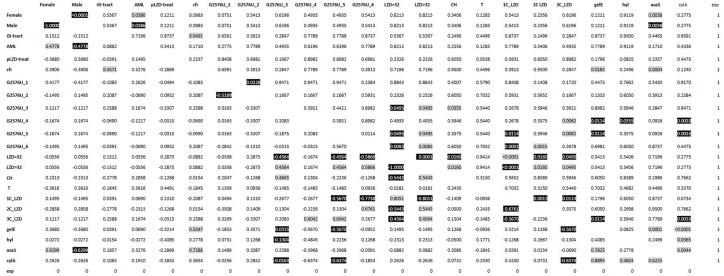
Significant positive and negative correlations between 14 measured parameters describing linezolid-resistant clinical *Enterococcus faecium* isolates. Legend: Values highlighted on grey background are statistically significant (p<0.05) and positively correlated. Values distinguished on black background are negatively correlated. Values are calculated using linear correlation statistic and uncorrected p-values (Past software v. 3.25). GI- tract–gastro-intestinal tract; pLZD–patients previously treated with linezolid; AML–patients with acute myeloid leukemia; *cfr*–gene coding for 23S rRNA methyltransferase; LZD–linezolid; G2576U_1 e.t.c–mutation in copy number 1 e.t.c; 1C_LZD—mutated 1 copy e.t.c; genes of virulence factors: *esp*—gene encoding enterococcal surface protein; *asa1*—gene encoding aggregation substance; *cylA*- gene encoding cytolysin; *gelE*- gene encoding gelatinase; *hyl*- gene encoding hyalorunidase.

The relationship between clinical, microbiological and genetic factors was depicted based on statistical data obtained (Fig 3).

**Fig 4:**
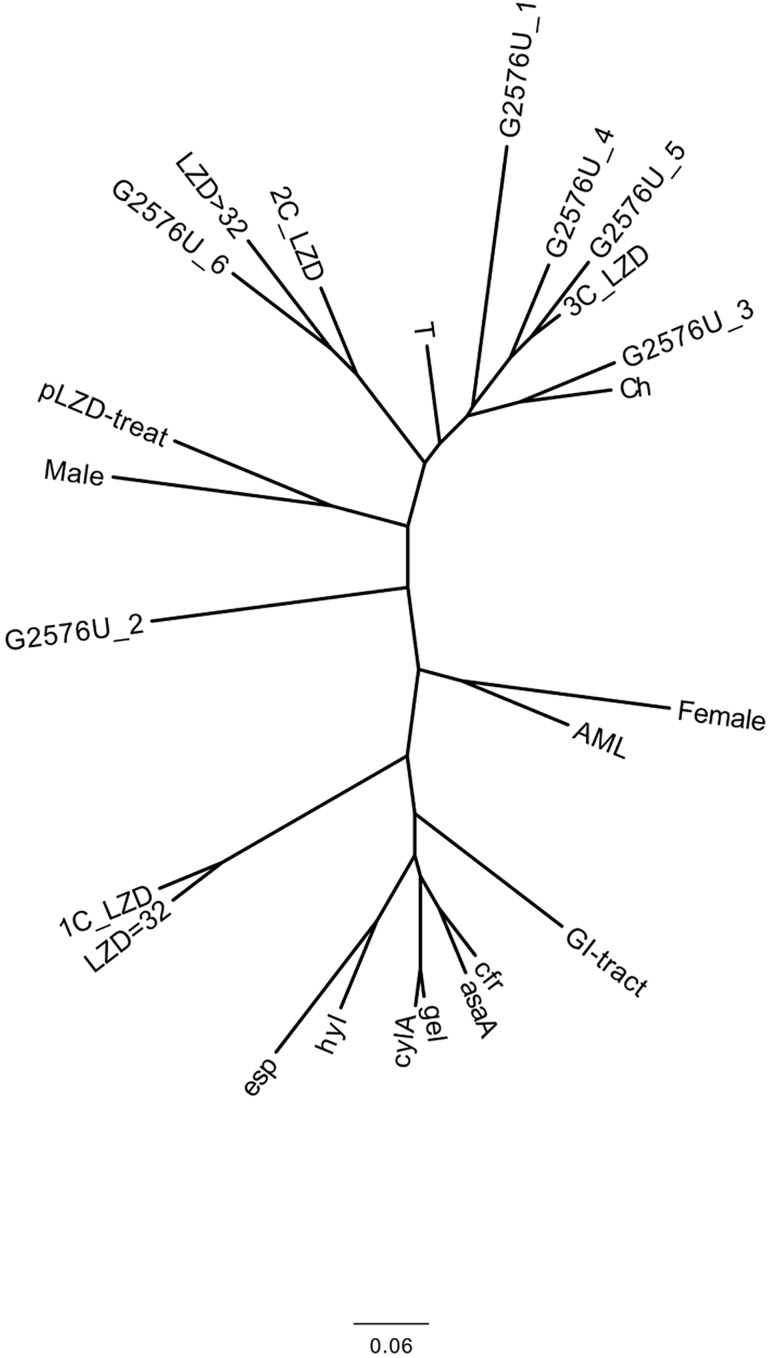
Pseudo-phylogenetic tree presenting similarity between the tested parameters. The correlation was examined between: virulence genes, resistance to antibiotics, location of G2576U mutation in six *rrn* operons, frequency of acute myeloid leukemia disease among patients, and gender of the patients. The tree was constructed by using the neighbor-joining method from 0–1 matrix. Description of parameters analyzed like in Fig 3.

The occurrence of the *cyl*A and *asa*1 gene in relation to the *gel*E gene was found statistically significant (p-value: <0.0001 and 0.0001, respectively). The *cyl*A gene is statistically significant in relation to the *hyl* and *asa*1 gene (p-value: 0.0365 and 0.0044, respectively), while the *asa*1 and *gel*E are statistically significant (p-value: 0.0004 and 0.0183) in relation to transferable oxazolidinone resistance gene (*cfr*). All virulence factors are closely adjacent to each other on the dendrogram (Fig 4) together with the *cfr* gene, which may suggest the same location on mobile elements and horizontal gene transmission.

It was also observed that the *cfr* gene is statistically significant (p-value: 0.0491) for gastrointestinal isolates. On the dendrogram all these virulence factors are closely adjacent to each other, forming one cluster. Hence it may be concluded that virulence and presence of multi-resistance *cfr* gene entails additional risk in case of colonization with LZD^R^ *E*. *faecium* strains or carrying them in the GI tract. Occurrence of mutations in one copy (p-value: 0.0001) and positive correlation with MIC LZD^R^ = 32 μg/mL are statistically significant. Positive correlation with more than one G2576U mutation was revealed for LZD^R^>32. If 3 mutated copies are present, it is most often a mutation in copies C1, C4 and C5. C6 mutation was found in isolates which had two mutated copies. In such case MIC exceeded 32 μg/mL. It is possible that two different mechanisms of resistance to linezolid, namely G2576U point mutation in the 23S rRNA subunit and the *cfr* gene encoding rRNA methyltransferase, which modifies the adenine residue at position 2503 in the V domain of 23S rRNA and additionally has effect on resistance to oxazolidinones. That is why additional mutation may increase resistance (e.g. in case of isolate 4 MIC amounted to 96 μg/mL with G2576U point mutation in one copy of the gene).

## Conclusions and summary

Selective pressure exerted by antibiotics on human microbiota is the major reason for the increase in the number of nosocomial infections caused by *E*. *faecium*. In case of enterococci this phenomenon is associated with common use of cephalosporins to which these microorganisms are naturally resistant, which in turn leads to a growing number of infections caused by these bacteria. In our hospital these are mainly *E*. *faecium* strains with VanA phenotype that are highly resistant to vancomycin and teicoplanin. Furthermore, treatment of infections caused by VRE with linezolid resulted in development of strains resistant to this antibiotic.

A specific conjugation of resistance to glycopeptides and linezolid took place, as evidenced by the fact that only 1/19 of *E*. *faecium* strains resistant to linezolid remained susceptible to glycopeptides. In such a case, earlier administration of linezolid to the patient as well as treatment with glycopeptides (vancomycin, teicoplanin) and possibly with carbapenems (imipenem) become factors in fostering colonization and infections with strains resistant to linezolid. The reason being the long-term antibiotic therapy, which eliminates commensal GI tract microbiota allowing multi-resistant bacteria to occupy a convenient ecological niche. This is supported by the observation that half of the patients, from whom LZD^R^EF were isolated, did not receive linezolid in the past. Patients are a reservoir of LZD^R^EF strains which may be responsible for spreading of multi-drug resistant enterococci in the hospital setting if appropriate isolation procedures are not followed. It should be noted that these microorganisms may be transmitted from carriers to the hospital setting and to other patients. This fact may be demonstrated by relatively close genotypic relationship between specific strains, even though they were isolated from different patients at a one- or two-month interval. The scenario also involves endogenous (commensal) microbiota being subjected to aggressive antibiotic therapy and chemotherapy. During such treatments sensitive microbiota species are eradicated and only the ones that are most adapted to hospital setting survive. The isolation procedure for strains in case of patients re-admitted at the ward, who were infected/colonized with LZD^R^VREF in the past, is another important issue. Such patients should be isolated until a negative microbiological test result is obtained. An increasing role of enterococci results from resistance to numerous groups of antibiotics, including resistance to glycopeptides and oxazolidinones, and it is also a determinant of virulence.

In patients with blood cancers and undergoing invasive procedures colonization may result in a high risk of complications, e.g. urinary tract infection, endocarditis or sepsis, which is increased by bacterial virulence factors that foster colonization and invasion.

In summary, typing methods with high discriminatory power are an excellent tool to determine epidemic relationships between strains; they also allow for detecting infections with endemic strains or suggest colonization (presence of the carrier). However, the same genotype determined by genetic typing is not equivalent to presence of the same mutated copy of the 23S rDNA gene. Quick methods based on HRM-real-time PCR applied in our study may be used to detect the basic G2576T mutation in the 23S rDNA, which most often correlates with increased resistance to linezolid. It very precisely detects point mutations, dividing the strains into relevant genotype groups. Furthermore, the traditional PCR-RFLP method allows identification of the corresponding *rrn* operon. Most probably the G2576T mutation may spread by way of conversion to other copies of 23S rDNA. Elevated MIC usually but not always correlate with increased number of mutated genes. This maybe further caused by the *cfr* gene being carried on plasmids or chromosomes, as it introduces a different mechanism of resistance to linezolid. We also observed that the *cfr* gene strongly correlates with the gastrointestinal environment, which indicates that enterococci carrying this gene may pose a risk of resistance to linezolid unrelated with the G2576U mutation being developed and may pose a risk of gene transmission within the remaining gastrointestinal microbiota. In addition, virulence factors strongly correlate with the *cfr* gene but not within the bacteria colonizing the GI tract, although a tendency towards the occurrence of linezolid-resistant enterococci rich in virulence factors in the GI tract may be observed. Additionally, linezolid-resistant strains may develop during treatment with this drug due to selective pressure; patients may become carriers of LZD^R^EF strains if they were previously treated with linezolid, which is why certain patient groups should be monitored for the multi-drug resistance of their gastrointestinal microbiota. On the other hand, absence of prior treatment with linezolid does not exclude the possibility that the patient could have been colonized with LZD^R^VREF strains which in the absence of competitive microbiota quickly settled ecological niches favourable for them and pose a risk for the patient. Risk factors for patients along with mechanisms with impact on development of the LZD^R^EF strains are summarized in S4 Fig.

Overall in our opinion, each hospital should have a strategic plan developed to detect, prevent and control infections and colonizations with the LZD^R^VREF strains.

## Supporting information

S1 Fig Hypothesis LZD^R^EF transmission between patients and hospital environment. (TIF)

S2 Fig Hypothesis of the origin colonization/infection of LZD^R^EF. Legend: LZD—linezolid; LZD^R^EF–*Enterococus faecium* with resistance to linezolid; LZD^S^EF—*Enterococus faecium* sensitive to linezolid.
(TIF)

S3 Fig Representative results of detection of G2576T mutation for *E*. *faecium* isolates using PCR-RFLP/NheI. M- the molecular DNA size marker 50–1000 bp (GeneRuler 50bp DNA ladder, ThermoScientific). K1 –wild strains without mutation after digestion of NheI enzyme; K2 –clinical strain with G2576T mutation without digestion. 1–13 representative isolates after digestion with NheI enzymes.
(TIF)

S4 Fig Summary of risk factors for the patient (A) and mechanisms of acquiring resistance to linezolid (B).
(TIF)

S1 Table Genes and oligonucleotides used for amplification. (DOCX)

## Abbreviations

LZD^R^VREF: linezolid-resistant vancomycin-resistant *Enterococcus faecium*
HLAR: High Level Aminoglycoside Resistance
VRE: Vancomycin-Resistant Enterococcus
LZD^R^EF: linezolid-resistant *Enterococcus faecium*
