# Supplementary figures and images for: Linezolid-resistant Enterococcus faecium strains isolated from one hospital in Poland –commensals or hospital-adapted pathogens?
Source: PLoS One. 2020 May 26;15(5):e0233504. doi: 10.1371/journal.pone.0233504 (PMC7250452; doi:10.1371/journal.pone.0233504)

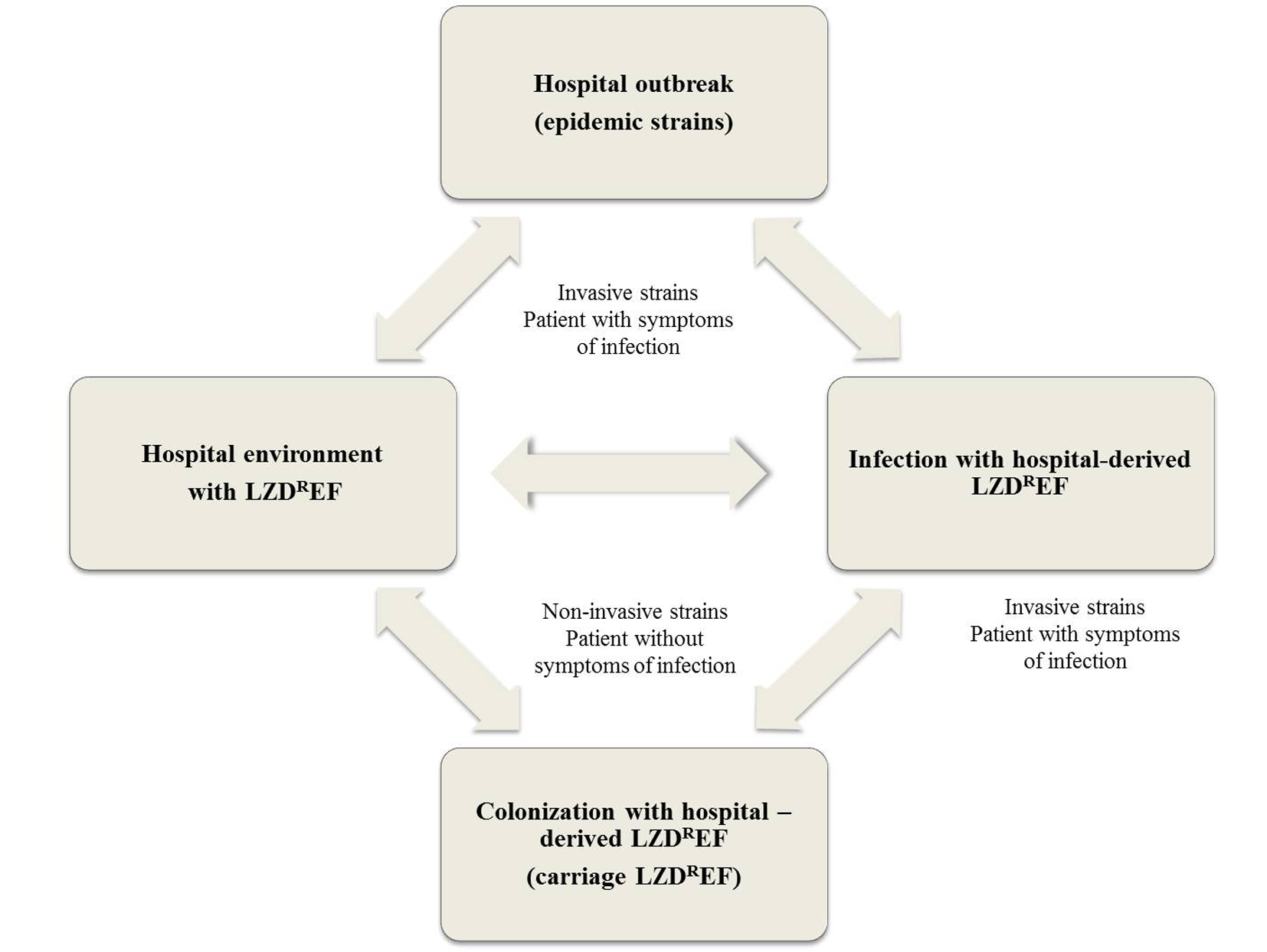

Supplement: S1 Fig — (TIF) [file pone.0233504.s001.tif]

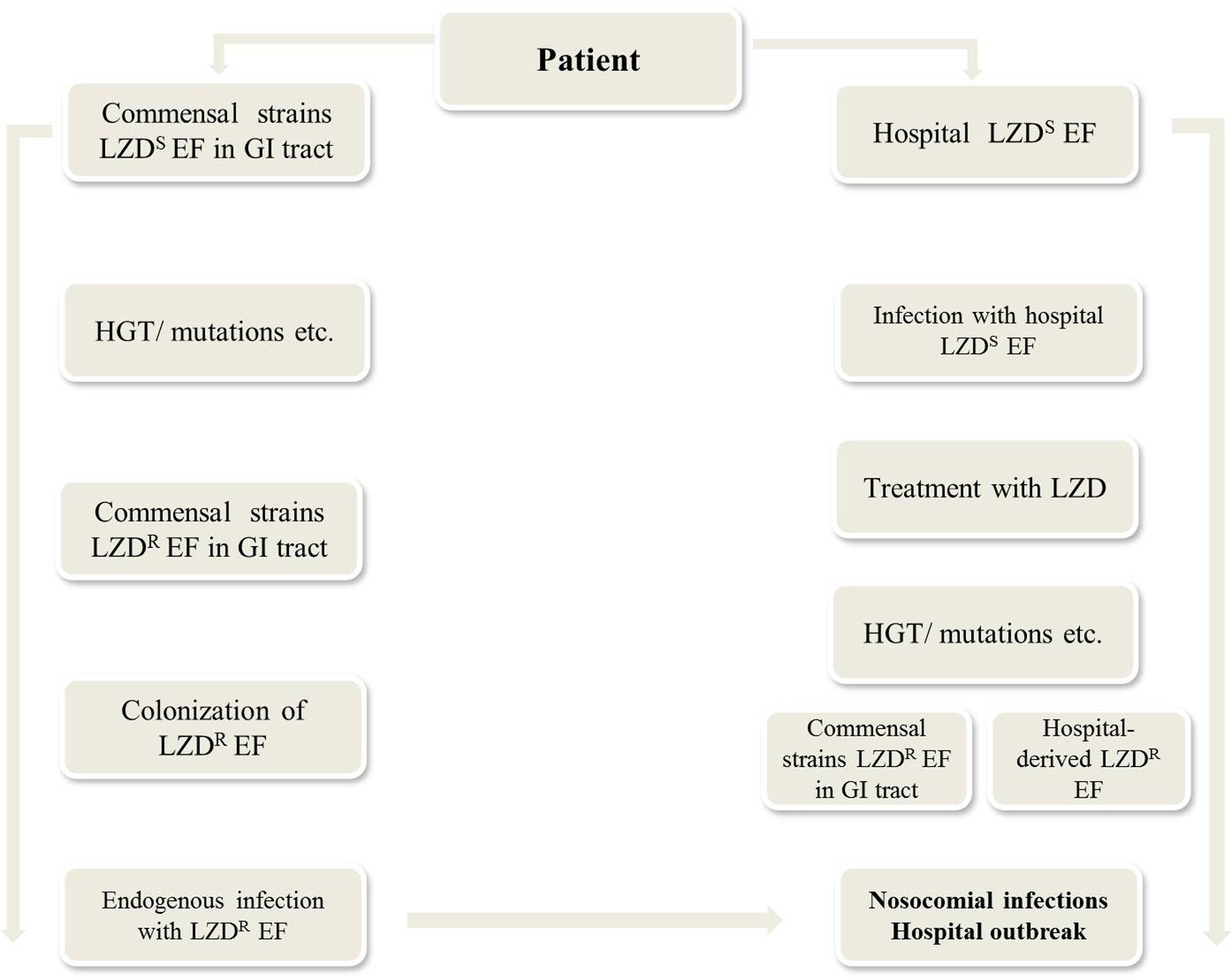

Supplement: S2 Fig — Legend: LZD—linezolid; LZDREF–Enterococus faecium with resistance to linezolid; LZDSEF—Enterococus faecium sensitive to linezolid. (TIF) [file pone.0233504.s002.tif]

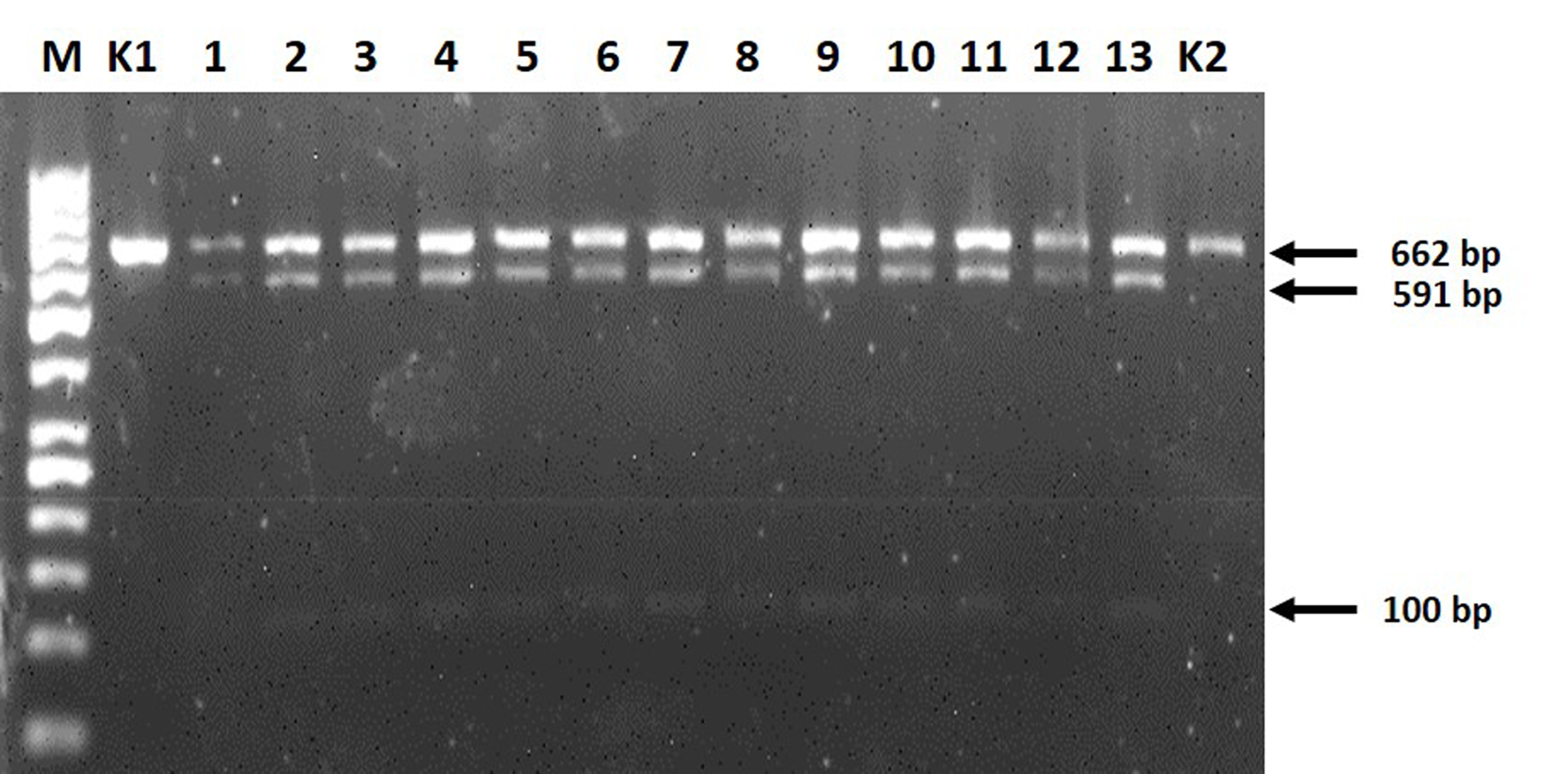

Supplement: S3 Fig — M- the molecular DNA size marker 50–1000 bp (GeneRuler 50bp DNA ladder, ThermoScientific). K1 –wild strains without mutation after digestion of NheI enzyme; K2 –clinical strain with G2576T mutation without digestion. 1–13 representative isolates after digestion with NheI enzymes. (TIF) [file pone.0233504.s003.tif]

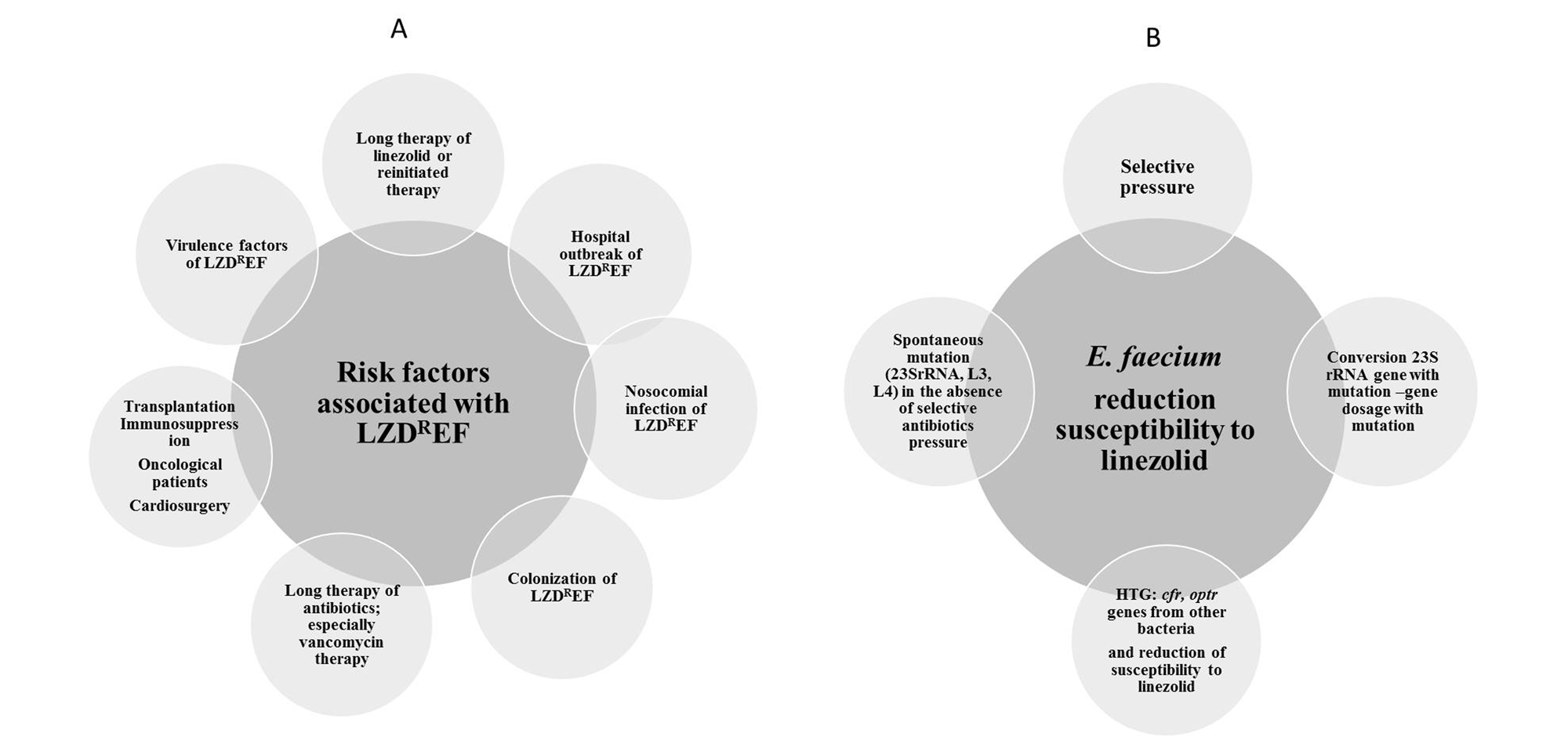

Supplement: S4 Fig — Summary of risk factors for the patient (A) and mechanisms of acquiring resistance to linezolid (B). (TIF) [file pone.0233504.s004.tif]
