# Supplementary material for: Linezolid-resistant Enterococcus faecium strains isolated from one hospital in Poland –commensals or hospital-adapted pathogens?
Source: PLoS One. 2020 May 26;15(5):e0233504. doi: 10.1371/journal.pone.0233504 (PMC7250452; doi:10.1371/journal.pone.0233504)
Supplement: S1 Table — (DOCX) [file pone.0233504.s005.docx]

**S1 Table. Genes and oligonucleotides used for amplification.**

| **Gene** | **Name oligonucleotide** | **Oligonucleotide sequence**  **(5’→3’)** | **Size of product (bp)** | **Reference** |
| --- | --- | --- | --- | --- |
| **PCR Melting Profile method** | | | | |
|  | aEcoHELP | AATTGTCGACGTTGG |  | in this study |
|  | aEcoLIG | CTCACTCTCACCAACGTCGAC C |  |  |
|  | Primer EcoMP | CTCACTCTCACCAACGTCGACAATT |  |  |
| **PCR/RFLP** | | | | |
| 23S rDNA | SfLR2100 | CGGTGAAATTTTAGTACCTGTGAAGATG | 662 | [47] |
|  | SfLr2 | GTCCATCCCGGTCCTCTCG |  |  |
| **real-time PCR** | | | | |
| 23SrDNA | For23 | GGTGGTTCCGCATGG | 196 | In this study |
|  | 23UniR2 | CGTTCTGAACCCAGCT |  |  |
| **resistance gene to antibiotics** | | | | |
| *van*A (vancomycin resistance A type) | vanAF | TTGGGGGTTGCTCAGAGGAG | 931 | [44] |
|  | vanAR | CTTCGTTCAGTACAATGCGG |  |  |
| *acc*(6’) - *aph*(2’’)  (HLGR high-level gentamicin resistance) | acphFIL | GATTTGCCAGAACATGAATTACACGA | 156 | [44] |
|  | acphRIL | CATAACCACTACCGATTATTTCAAT |  |  |
| *cfr* | cfr-fw | TGAAGTATAAAGCAGGTTGGGAGTCA | 746 | [45, 46] |
|  | cfr-rv | ACCATATAATTGACCACAAGCAGC |  |  |
| *optr*A | FoptrA | AGGTGGTCAGCGAACTAA | 1395 | [30] |
|  | RoptrA | ATCAACTGTTCCCATTCA |  |  |
| **virulence factor genes** | | | | |
| *cyl*A | TE17 | TGGATGATAGTGATAGGAAGT | 517 | [38, 39] |
|  | TE18 | TCTACAGTAAATCTTTCGTCA |  |  |
| *hyl* | hyl1 | ACAGAAGAGCTGCAGGAAATG | 276 | [38] |
|  | hyl2 | GACTGACGTCCAAGTTTCCAA |  |  |
| *gel*E | gel11 | TATGACAATGCTTTTTGGGAT | 213 | [38] |
|  | gel12 | AGATGCACCCGAAATAATATA |  |  |
| *asa*1 | agg1 | GGTGCCACAATCAAATTAGG 3' | 379 | [38, 39] |
|  | agg2 | GATTCTTCGATTGTGTTGGTAAACG 3' |  |  |
| *esp* | esp11 | TTGCTAATGCTAGTCCACGACC 3' | 954 | [39] |
|  | esp12 | GCGTCAACACTTGCATTGCCGAA 3' |  |  |
| *ddl* | ECIUMF | CGC AGA GCA TGA AGT GTC CA 3' | 557 | [43] |
|  | ECIUMR2 | CTT CTC GGT TTT CTG CTT TTG TA 3' |  |  |
| **copies of the 23S rDNA gene** | | | | |
| universal 23S rDNA | SfLR2100 | CGGTGAAATTTTAGTACCTGTGAAGATG |  | [47] |
| copy 1/ 23S rDNA | Efrev1/C1 | TAATCGAACGATTTGCACCA | 1223 | [48] |
| copy 2/ 23S rDNA | Efrev2 /C2 | AGCAGGAATTGAACCCACAC | 1833 |  |
| copy 3/ 23S rDNA | Efrev3/C3 | TTGCCGAAATGAGTCTGAAA | 1346 |  |
| copy 4/ 23S rDNA | Efrev4 /C4 | CATCATTTTAAGAATAGGAAAGGACA | 1337 |  |
| copy 5/ 23S rDNA | Efrev5 /C5 | ACTCGAACCATCGACCTCAC | 2590 |  |
| copy 6/ 23S rDNA | Efrev6 /C6 | GAGGTCTGCCATTTCAATCC | 2175 |  |
